# Inhibition of miR‐34a‐5p can rescue disruption of the p53‐DAPK axis to suppress progression of clear cell renal cell carcinoma

**DOI:** 10.1002/1878-0261.12545

**Published:** 2019-08-24

**Authors:** Zhi‐Fei Jing, Jian‐Bin Bi, Zeliang Li, Xiankui Liu, Jun Li, Yuyan Zhu, Xiao‐Tong Zhang, Zhe Zhang, Zhenhua Li, Chui‐Ze Kong

**Affiliations:** ^1^ Department of Urology First Hospital of China Medical University Shenyang Liaoning China; ^2^ Institute of Urology China Medical University Shenyang China

**Keywords:** clear cell renal cell carcinoma, DAPK, miR‐34a‐5p, p53

## Abstract

DAPK, a transcriptional target of the p53 protein, has long been characterized as a tumor suppressor that acts as a negative regulator in multiple cellular processes. However, increasing studies have suggested that the role of DAPK may vary depending on cell type and cellular context. Thus far, the expression and function of DAPK in clear cell renal cell carcinoma (ccRCC) remain ambiguous. Since ccRCC behaves in an atypical way with respect to p53, whether the p53‐DAPK axis functions normally in ccRCC is also an intriguing question. Here, tissue specimens from 61 ccRCC patients were examined for DAPK expression. Functional studies regarding apoptosis, growth, and migration were used to determine the role of DAPK in renal cancer cells. The validity of the p53‐DAPK axis in ccRCC was also determined. Our study identified DAPK as a negative regulator of ccRCC, and its expression was reduced in certain subgroups. However, the p53‐DAPK axis was disrupted due to upregulation of miR‐34a‐5p under stressed conditions. miR‐34a‐5p was identified as a novel repressor of DAPK acting downstream of p53. Inhibition of miR‐34a‐5p can correct the p53‐DAPK axis disruption by upregulating DAPK protein and may have potential to be used as a therapeutic target to improve outcomes for ccRCC patients.

**Abbreviations**
ccRCC: clear cell renal cell carcinoma
EdU: 5‐ethynyl‐2'‐deoxyuridine
IHC: immunohistochemistry
PARP: poly ADP‐ribose polymerase
TCGA: The Cancer Genome Atlas
TUNEL: terminal deoxynucleotidyl transferase dUTP nick end labeling

## 1 Introduction

Renal cell carcinoma (RCC) is the second most common cancer in the urological system and accounts for approximately 3% of malignant neoplasms worldwide (Rini *et al.*, 2009). Clear cell RCC is the most common histologic subtype of RCC characterized by its resistance to conventional chemotherapy and radiotherapy (Jemal *et al.*, 2007; Lopez‐Beltran *et al.*, 2006). Although various possible mechanisms responsible for this resistance have been reported (Zhou *et al.*, 2014), the role of p53 in this process awaits further elucidation. Thus far, TP53 is the most studied tumor suppressor gene found to be mutated in approximately 50% of human cancers (Vousden and Lane, 2007). The p53 protein mainly functions as a transcription factor to induce cell cycle arrest in response to cellular or genotoxic stress, or to promote apoptosis when the damage was too severe to be repaired (Jackson and Bartek, 2009; Riley *et al.*, 2008). Intriguingly, several lines of evidence indicated that p53 is rarely mutated in ccRCC (Noon *et al.*, 2012); however, upregulation of wild‐type p53 protein failed to inhibit disease progression or improve the prognosis of ccRCC patients (Mombini *et al.*, 2006; Noon *et al.*, 2010). We therefore believed that the p53 pathway is suppressed in ccRCC and that the factor responsible for this suppression lies downstream of p53.

DAPK (also known as DAPK1) is a p53 downstream transcriptional target. DAPK is a 160‐kDa serine/threonine kinase, which was originally isolated in an unbiased antisense‐based genetic screen for genes whose protein products were necessary for interferon‐γ‐induced death in HeLa cells (Deiss *et al.*, 1995). The ability of p53 to induce DAPK transcription was originally described by Martoriati in Saos‐2 and MEF cells (Martoriati *et al.*, 2005). Later, studies showed that p53 also induced DAPK transcription in CNE1 cells (Luo *et al.*, 2011). Recent studies found that DAPK was involved in various cellular processes such as apoptosis, autophagy, cell motility, and anoikis (Cohen *et al.*, 1999; Gozuacik *et al.*, 2008; Guenebeaud *et al.*, 2010; Inbal *et al.*, 2002; Lin *et al.*, 2010). DAPK is thus characterized as a negative regulator of cancer. The expression of DAPK is reduced in the majority of cancers due to the methylation of the DAPK promoter (Kissil *et al.*, 1997; Mittag *et al.*, 2006). However, findings regarding the expression of DAPK in ccRCC and adjacent non‐neoplastic renal tissues are ambiguous (Christoph *et al.*, 2007; Wethkamp *et al.*, 2006; Yukawa *et al.*, 2004), although high CpG island methylation was detected in the DAPK gene promoter region (Christoph *et al.*, 2006; Wethkamp *et al.*, 2006). In addition, previous studies did not address the function of DAPK in ccRCC and whether the p53‐DAPK axis functions normally in ccRCC remains unknown.

MicroRNA (miRNA or miR) is a class of endogenously expressed noncoding regulatory RNAs that contain 21–25 nucleotides. miRNAs can bind to the 3'‐UTRs of mRNAs in a sequence‐specific manner, resulting in mRNA degradation or translation suppression (Croce, 2009; He and Hannon, 2004). Abnormal expression of various microRNAs has been associated with carcinogenesis by functioning either as a tumor suppressor or as an oncogene (Zhang *et al.*, 2007). Recently, it has been shown that miRNAs are involved in the progression and metastasis of RCC (Chow *et al.*, 2010; Gottardo *et al.*, 2007; White *et al.*, 2011).

The objective of this study was to determine the expression of DAPK and its function in apoptosis, growth, and migration in ccRCC. We further investigated the validity of the p53‐DAPK axis in ccRCC and determined how miRNAs interacted with p53 and DAPK in ccRCC.

## 2 Materials and methods

### 2.1 Cell culture and transfection

Human HK‐2, ACHN, A498, CAKI‐1, 786‐O, 769‐P, OS‐RC‐2, and 293T cell lines were obtained from the Cell Bank of Shanghai Institute of Cell Biology (Chinese Academy of Medical Science, Shanghai, China). 786‐O, 769‐P, and OS‐RC‐2 were maintained in RPMI 1640 (HyClone, Logan, UT, USA) supplemented with 10% FBS (HyClone) at 37 °C with 5% CO_2_. ACHN and A498 were cultured in MEM (HyClone) with 10% FBS. CAKI‐1 was cultured in McCoy'5a Medium Modified (HyClone) with 10% FBS. HK‐2 was cultured in Dulbecco’s modified Eagle’s medium (DMEM)/F12 (HyClone) medium with 10% FBS. 293T cells were cultured in DMEM (HyClone) with 10% FBS. Transfection was carried out using Lipofectamine 3000 Reagent (Invitrogen, Carlsbad, CA, USA). In the MOCK group, only transfection reagents were added. Empty vectors (pCMV3‐untagged) were used as control. Stable DAPK knockdown cell lines were generated by lentiviral infection using shRNA against DAPK. Stable DAPK knockdown cell lines were then maintained by puromycin selection (2 µg·mL^−1^). Stable DAPK overexpression cell lines were established by transfection of pCMV3‐Hygro‐DAPK expression plasmids. Forty‐eight hours later, cells were selected using fresh medium containing 100 μg·mL^−1^ Hygromycin B (Roche, Mannheim, Germany).

### 2.2 Reagents and antibodies

Z‐VAD‐FMK was purchased from Selleck (Selleckchem, Houston, TX, USA). Nutlin‐3, tenovin‐1, and doxorubicin were commercially available from MedChem Express (Shanghai, China). The DAPK siRNA and p53 siRNA were purchased from GenePharma (Shanghai, China). LV‐DAPK1‐RNAi was purchased from GeneChem (Shanghai, China). The miR‐34a‐5p mimics, inhibitor, antagomiR, and negative control duplex were synthesized by Shanghai GenePharma. The antibodies and related reagents used in this study were obtained as follows: anti‐DAPK1 (3008S for immunoblot; Cell Signaling, Danvers, MA, USA), anti‐poly ADP‐ribose polymerase (PARP) (9532S; Cell Signaling), anti‐E‐cadherin (3195T; Cell Signaling), anti‐vimentin (5741T; Cell Signaling), anti‐pDAPK‐Ser308 (D4941; Sigma, St. Louis, MO, USA), anti‐GAPDH (G9545; Sigma), anti‐p53 (sc‐126; Santa Cruz, Dallas, TX, USA), Apoptosis Western Blot Cocktail (ab136812; Abcam, Cambridge, MA, USA), anti‐Ki67(ab15580; Abcam), anti‐DAPK1 [BA3712‐1 for immunohistochemistry(IHC); Boster Biological Technology, Wuhan, China], and anti‐N‐cadherin(CDH2) (ab76011; Abcam).

### 2.3 Patients

Sixty‐one ccRCC specimens and matched non‐neoplastic renal tissues were obtained from the Urology Department, First Hospital of China Medical University (Shenyang, China), between September 2015 and December 2016. Freshly resected tissue specimens were kept at −80 °C for quantitative PCR (qPCR) and immunoblot and in 10% formalin for IHC until use. All tumor specimens were from primary ccRCC and were reviewed by two pathologists. Grading of the renal tumor was based on the Fuhrman nuclear grading system, and staging was based on the principles outlined by the American Joint Committee on Cancer 2010. The Ethics Committee of China Medical University approved this study according to the Declaration of Helsinki. Informed written consent was obtained from all individual participants included in the study.

### 2.4 Cell proliferation assays

Cell proliferation was measured by CCK‐8 assays, 5‐ethynyl‐2'‐deoxyuridine (EdU) staining, and colony formation assays. In CCK‐8 assays, 2000 cells were seeded in 96‐well plates containing 100 μL of medium per well and incubated at 37 °C. CCK‐8 reagents (Dojindo, Tokyo, Japan) were added and coincubated for 1 h at different time points. The absorbance value was then measured at a wavelength of 450 nm with a microplate reader (Model 680; Bio‐Rad laboratories, Hercules, CA, USA). The results represent the average of four independent replicates. For EdU staining assays, EdU (10 mm) was added to the culture medium and incubated for 6–12 h. The cells were then fixed and stained with DAPI. The number of EdU‐positive cells was observed under a fluorescence microscope. EdU‐positive cells were counted in five randomly selected fields. For the colony formation assay, 300 cells per well were plated in six‐well plates, and 10 days after seeding, cell colonies were stained with crystal violet and counted.

### 2.5 Apoptosis assays

Cell apoptosis was measured by flow cytometry using the Annexin V‐FITC/PI Apoptosis Detection Kit (BD556547; BD Biosciences, Franklin Lakes, NJ, USA) according to the manufacturer's protocols. At 36–48 h post‐transfection, cells were harvested and incubated with FITC‐conjugated Annexin V and propidium iodide for 15 min at room temperature in the dark. Then, samples were analyzed on a FACScan flow cytometer (BD Biosciences).

### 2.6 Hoechst 33342 nuclear staining analysis

Briefly, the ACHN and 786‐O cancer cells were plated in 6‐well plates and then transfected with DAPK expression vectors or empty vectors. Forty‐eight hours later, the cells were stained with Hoechst 33342 (Beyotime, Shanghai, China) for 10 min and then observed by fluorescence microscopy (IX71; Olympus, Tokyo, Japan). Cells with apoptotic changes were counted in five randomly selected fields.

### 2.7 Migration assay

The ability of ACHN and 786‐O cells (50 000 cells/well) to migrate was determined by both Boyden chambers (Corning Costar, Corning, NY, USA) and the xCELLigence RTCA system. Cells that migrated to the lower side of the Boyden chambers were stained with crystal violet and counted. The numbers of migrated cells were counted in five randomly selected fields for each chamber. RTCA was also used to monitor the migration of cells in real time as determined with the use of the xCELLigence system cell invasion/migration (CIM) plates. Medium containing 10% FBS (165 μL per well) was added to the lower chamber of the CIM plates, while 30 μL per well of medium without FBS was added to the upper chamber. After incubation at 37 °C for 1 h, baseline background levels were recorded. The ACHN or 786‐O (50 000/well) cell suspensions without FBS were added to the upper chamber. Real‐time dynamic analysis of migration was recorded at 10‐min intervals over a 10‐h period.

### 2.8 Wound‐healing assay

For the wound‐healing assay, cells were seeded in six‐well plates at a density of 10^6^ cells/well. An artificial scratch was made using a 1‐mL pipette tip when the density reached approximately 95%. After washing three times with PBS, the medium was replaced with fresh medium. Images were captured using an inverted microscope (EVOS XL system, AMEX1200; Life Technologies Corp, Bothell, WA, USA) at different time points. The wound‐healing rate was analyzed by imagej (1.51s), and migration curves were drawn using the following formula: the area of the wound closed = the wound area at the beginning − the wound area at different time points.

### 2.9 Dual‐luciferase reporter assay

The pGL3 luciferase reporter constructs with the genomic region of human DAPK that contain the miR‐34a‐5p binding site were constructed by GenePharma, and pGL3 reporters with mutant miR‐34a‐5p binding sites and pGL3 reporters without binding sites were used as a control. The wild‐type or mutant constructs (2.0 μg/well in 6‐well plates) of DAPK were transfected into 293T cells together with miR‐34a‐5p mimics (50 nm) or the negative control (50 nm). A Renilla luciferase reporter plasmid was used as an internal reference. Forty‐eight hours after transfection, the cells were lysed in passive lysis buffer, and Firefly and Renilla luciferase activities were tested using the Dual‐Luciferase Reporter Assay System (Promega, Madison, WI, USA) according to the manufacturer's instructions. The firefly luciferase activity results were normalized to the Renilla activity results.

### 2.10 Immunohistochemistry

The specimens that were previously formalin‐fixed and paraffin‐embedded were sliced into 4‐μm sections and processed for deparaffinization and then rehydration. Antigen retrieval, suppression of endogenous peroxidase activity, and 10% BSA blocking were performed before primary antibody incubation. DAPK and Ki67 antibodies were used as primary antibodies overnight at 4 °C. The slides were subsequently incubated with peroxidase‐conjugated secondary antibody (ZSGB‐Bio, Beijing, China) for 90 min, and a solution of peroxidase‐labeled polymer, 2,4‐diaminobutyric acid, was used for signal development for 1 min. The sections were counterstained with hematoxylin followed by dehydrating and mounting.

### 2.11 RNA extraction and real‐time qPCR

Total RNA was extracted using the Eastep Super Total RNA Extraction Kit (Promega). miRNA was extracted using the miRcute miRNA Isolation Kit (Tiangen Biotech, Beijing, China). First‐strand cDNA was synthesized with Prime Script RT Master Mix (TaKaRa, Tokyo, Japan) or Mir‐X™ miRNA First‐Strand Synthesis Kit (TaKaRa). qPCR was conducted using SYBR Green PCR Master Mix (TaKaRa) on a LightCycler 480 Real‐Time PCR instrument (Roche, Indianapolis, IN, USA). The primers used for target genes were purchased from Sangon Biotech (Shanghai, China). The sequences of the primers were as follows (in the 5'–3' orientation): DAPK forward, 5'‐AGAAATTCAAGAAGTTTGCAG‐3'; DAPK reverse, 5'‐GTCTTCCTCATCCAGAGTAT‐3'; p53 forward, 5'‐ CCACCATCCACTACAACT C‐3'; p53 reverse, 5'‐AAACACGCACCTCAAAGC‐3'; p21 forward, 5'‐TAGCAGCGGAACAAGGAG‐3'; p21 reverse, 5'‐AAACGGGAACCAGGACAC‐3'; PUMA forward, 5'‐GACCTCAACGCACAGTACGA‐3'; PUMA reverse, 5'‐GAGATTGTACAGGACCCTCCA‐3'; Bax forward, 5'‐TGGCAGCTGACATGTTTTCTGAC‐3'; Bax reverse, 5'‐TCACCCAAC CACCCTGGTCTT‐3'; beta‐actin forward, 5'‐CATGTACGTTGCTATCCAGGC‐3'; beta‐actin reverse, 5'‐CTCCTTAATGTCACGCACGAT‐3'; hsa‐miR‐34a‐5p, 5'‐CTGGCAGTGCTGGTTGT‐3'; beta‐actin and U6 were used as an internal control for mRNA and miRNA, respectively. Data are presented as relative quantification based on the calculation of $2^{-\Delta \Delta {\text{C}}_{t}}$.

### 2.12 Western blot

Cells were rinsed with cold PBS and homogenized with RIPA lysis buffer supplemented with 1 mm PMSF (Beyotime), and phosphatase inhibitor cocktail (MCE) was added according to the manufacturer’s recommendation. Afterward, 30 μg of whole cell lysates was separated by 4–20% SDS/PAGE and electrophoretically transferred onto a polyvinylidene fluoride membrane (Millipore, Temecula, CA, USA). The membrane was washed with TBST, blocked with 5% skim milk, diluted in TBST for 1 h, and then incubated with appropriate primary antibodies overnight at 4 °C. The membrane was washed and then incubated with HRP‐conjugated secondary antibodies for 1 h at 37 °C. Bands were visualized with the enhanced chemiluminescence reagents (TransGen Biotechnology, Beijing, China) and scanned using a DNR Microchemi imaging system. Protein quantification was conducted using imagej software.

### 2.13 Tumor xenografts in nude mice

To determine the effects of DAPK on renal cancer cells *in vivo*, DAPK‐overexpressing, sh‐DAPK, and control ACHN cells were harvested (5 × 10^6^ cells resuspended in 150 μL of Matrigel) and subcutaneously injected into the right flank of fifteen 4‐week‐old female BALB/c nude mice (five mice for each group). One week after implantation, tumor sizes were measured twice a week for 3 weeks. Tumor length (L) and width (W) were measured with a caliper, and tumor volumes were calculated using the formula (L × W^2^)/2. The animals were sacrificed, and the tumors were harvested for immunoblotting and IHC.

To investigate the impact of miR‐34a on ccRCC cells *in vivo*, 5 × 10^6^ cells resuspended in 150 μL of Matrigel were subcutaneously injected into the flank of 12 nude mice. One week after implantation, mice were randomized into four groups (*n* = 3 in each group). NC‐antagomiR, antagomiR‐34a, doxorubicin, and doxorubicin in combination with antagomiR‐34a were used to treat the mice. NC‐antagomiR and antagomiR‐34a (10 nmol diluted in 20 μL PBS) were injected intratumorally on days 7, 9, and 11, while doxorubicin (2 mg·kg^−1^) was injected via tail vein on days 14 and 17. Tumor sizes were monitored twice a week for 3 weeks. The mice were sacrificed on day 28, and the tumors were harvested for immunoblotting, IHC, and terminal deoxynucleotidyl transferase dUTP nick end labeling (TUNEL) assay.

For TUNEL assay, apoptotic cells in 4‐μm sections of paraffin‐embedded tumor samples were detected by TUNEL BrightRed Apoptosis Detection Kit (Vazyme Biotech, Nanjing, China) according to the manufacturer’s instructions and the samples were analyzed by fluorescence microscopy. The number of apoptotic cells per field was quantified in 5 randomly selected microscopic fields.

All procedures were performed in accordance with the Animal Care and Use Committee guidelines of China Medical University.

### 2.14 Statistics

The results are presented as the mean ± standard deviation. The *t*‐tests and ANOVA were used where appropriate. Correlations were calculated according to the Pearson correlation. All tests were performed using graphpad prism 7 GraphPad Software (La Jolla, CA, USA), and *P* < 0.05 was considered significant. All experiments were performed at least three times.

## 3 Results

### 3.1 DAPK expression was reduced in certain subgroups of clear cell renal cell carcinoma

**Figure 1:**
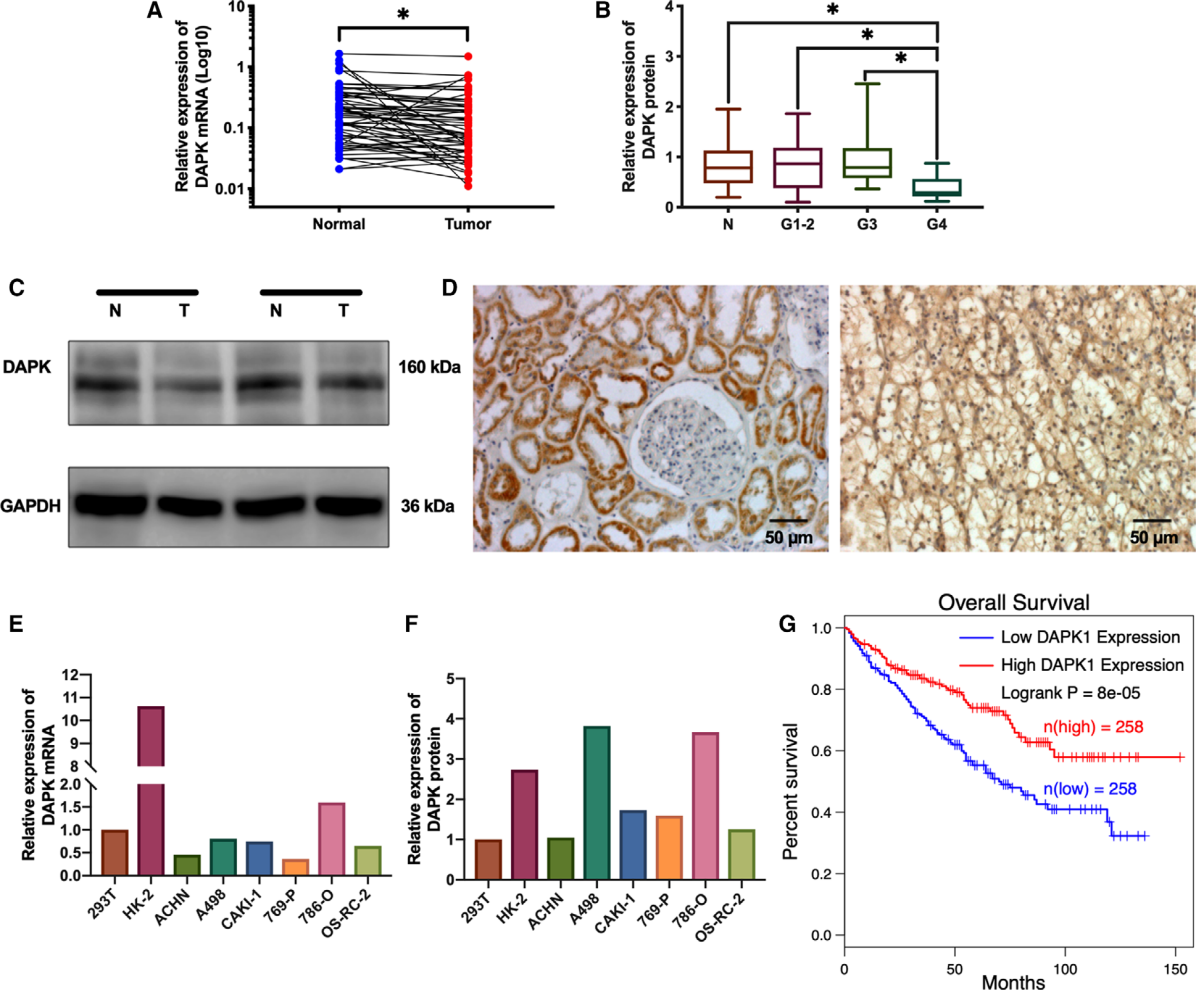
The expression of DAPK mRNA and DAPK protein in ccRCC tissue samples and renal cancer cell lines. (A) Relative expression of DAPK mRNA in normal renal tissues and paired ccRCC samples. *P(paired *t*‐test) < 0.05 (*n* = 61). (B) DAPK protein expression in normal renal tissues and ccRCC samples of different grades. *P(one‐way ANOVA) < 0.05. (C) Immunoblotting analysis of DAPK in normal renal tissues and grade 4 tumors. (D) The IHC detection of DAPK protein in normal renal tissues (left) and ccRCC (right). Scale bar, 50 μm. (E, F) Relative expression of DAPK mRNA and protein in 293T, HK‐2, and renal cancer cell lines. (G) TCGA data from GEPIA (http://gepia.cancer-pku.cn) indicated that low DAPK expression predicted poor prognosis of ccRCC patients.

To determine the expression of DAPK mRNA and DAPK protein in ccRCC, a total of 61 renal tumor tissues and paired normal renal tissues were collected and subjected to qPCR and immunoblotting. The patient characteristics are summarized in Table 1. Twelve pairs of tissue samples were further analyzed by IHC. The qPCR results showed that DAPK mRNA expression was lower in ccRCC specimens than in the normal counterparts, which is consistent with transcriptional data from The Cancer Genome Atlas (TCGA) (Figs 1A and S1A‐C). However, no significant difference in DAPK mRNA expression was observed between tumors of different stages or grades due to limited sample size in our study (Fig. S1D,E).

**Table 1:** Patient characteristics.

No. of patients	61
Male	38 (62.3%)
Female	23 (37.7%)
Age (years), mean ± SD	62.5 ± 8.8
Side	
Left	32 (52.5%)
Right	29 (47.5%)
Stage	
T1	23 (37.7%)
T2	29 (47.5%)
T3	9 (14.8%)
Grade	
G1/2	39 (63.9%)
G3	12 (19.7%)
G4	10 (16.4%)

Immunoblot analysis showed no significant difference between renal tumors and non‐neoplastic renal tissues (Fig. S1F). Renal tumors of different stages showed comparable DAPK protein expression (Fig. S1G). However, DAPK protein expression was lower in G4 tumors than in tumors of other grades and normal renal tissues (Fig. 1B,C).

Findings from IHC showed that DAPK protein expression was detected in both the nuclei and cytoplasm of clear cell renal tumor cells (Fig. 1D). Of the 12 tumor samples, three samples showed intense DAPK staining and the remaining tissue samples showed weak staining. In non‐neoplastic renal tissue samples, DAPK protein was mainly found in the cytoplasm of kidney tubular epithelial cells. No immunoreactivity was found in the glomeruli of the kidney. Nine of the normal renal tissues displayed intense DAPK staining, and the other three samples showed weak staining intensity.

The DAPK mRNA and protein expression levels in 293T, HK‐2, and renal cancer cell lines were also examined (Fig. 1E,F), and the DAPK mRNA expression correlated with DAPK protein expression in these cell lines.

Follow‐up data from the TCGA KIRC database showed that patients with relatively high DAPK mRNA expression had better prognosis than those with low DAPK expression (Fig. 1G).

### 3.2 DAPK promoted the apoptosis of renal cancer cells

**Figure 2:**
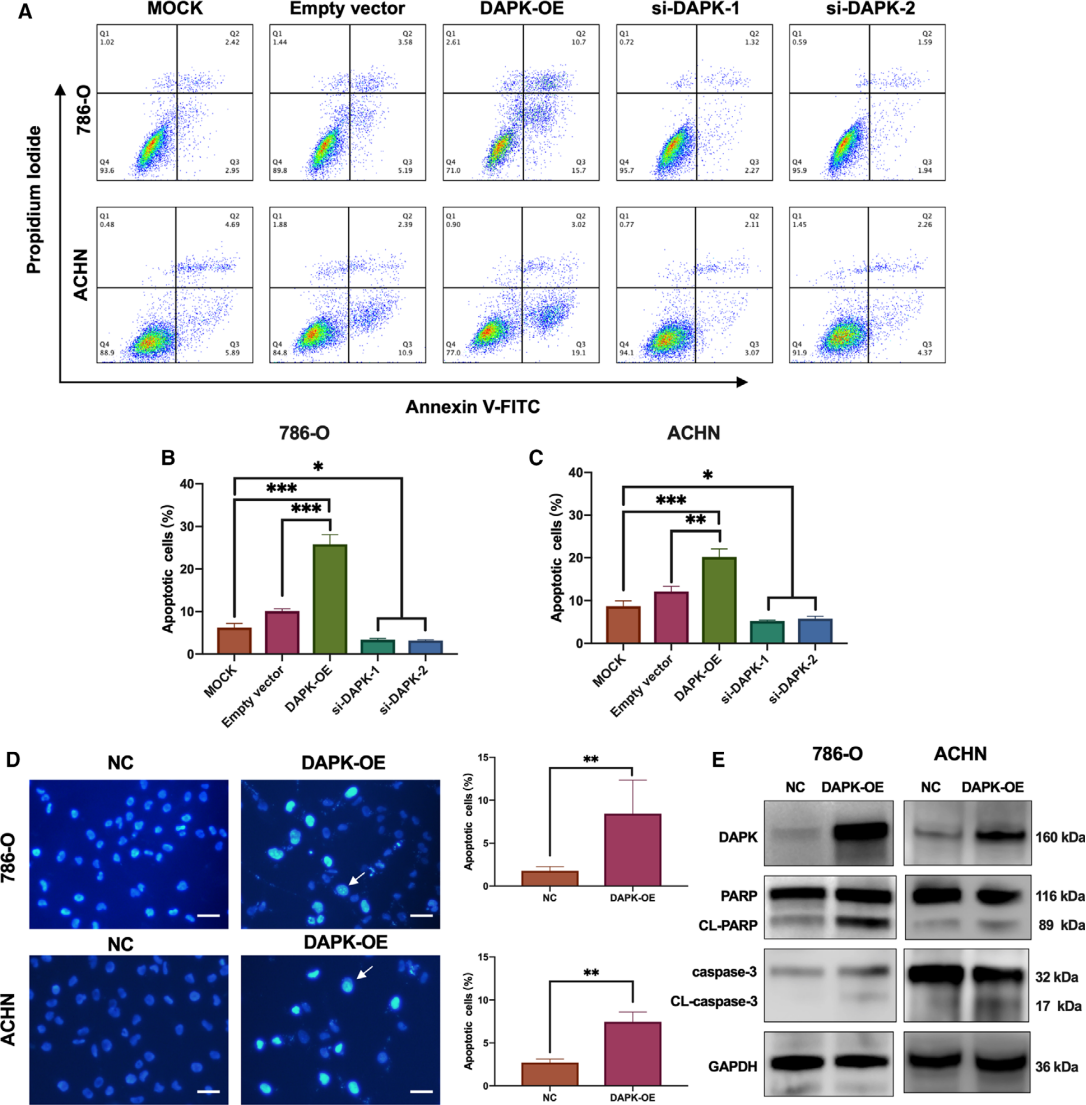
Analysis of DAPK‐mediated renal cancer cell apoptosis. (A) The DAPK‐mediated apoptosis of 786‐O and ACHN cells was analyzed by Annexin V‐FITC and propidium iodide staining followed by flow cytometry. In the MOCK group, Lipofectamine 3000 and P3000 were added, and in the empty vector group, pCMV3‐untagged vectors were transfected. (B, C). Percentages of cell apoptosis were shown as bar charts. *P(one‐way ANOVA) < 0.05, **P < 0.01, ***P < 0.001. Data are presented as mean ± SD (*n* = 3). (D). 786‐O and ACHN cells exhibited apoptotic changes 48 h after DAPK transfection, including condensed chromatin and fragmented nuclei, indicated by the white arrows. Scale bar, 40 μm. The percentage of apoptosis was quantified by calculating the number of apoptotic cells/total cells per high‐power field. **P(Student’s *t*‐test) < 0.01. Data are presented as mean ± SD (*n* = 5). (E) Immunoblotting analysis of cleaved PARP (CL‐PARP) and cleaved caspase‐3 (CL‐caspase‐3) induced by DAPK overexpression.

To determine the effect of DAPK on apoptosis of renal cancer cells, different renal cancer cell lines were used. DAPK expression vectors and DAPK siRNAs were transfected into renal cancer cell lines, and apoptosis was analyzed 36 h later. Findings from flow cytometry showed that DAPK overexpression induced apoptosis in 786‐O, ACHN, and 769‐P cells, which can be rescued by the addition of pan‐caspase inhibitor Z‐VAD‐FMK (20 μm), whereas DAPK knockdown reduced the proportion of apoptosis in 786‐O and ACHN cells (Figs 2A–C and S2A). We further used Hoechst 33342 nuclear staining to determine the impact of DAPK on apoptosis of 786‐O and ACHN cells (Fig. 2D). The nuclear staining showed that DAPK overexpression led to more cells with fragmented and intensely stained nuclei. Immunoblot analysis showed that cleaved caspase‐3 was hardly detected in 786‐O and ACHN cells under normal culture conditions; however, DAPK overexpression promoted the cleavage of both caspase‐3 and PARP (Fig. 2E).

### 3.3 DAPK inhibited the proliferation of renal cancer cells

**Figure 3:**
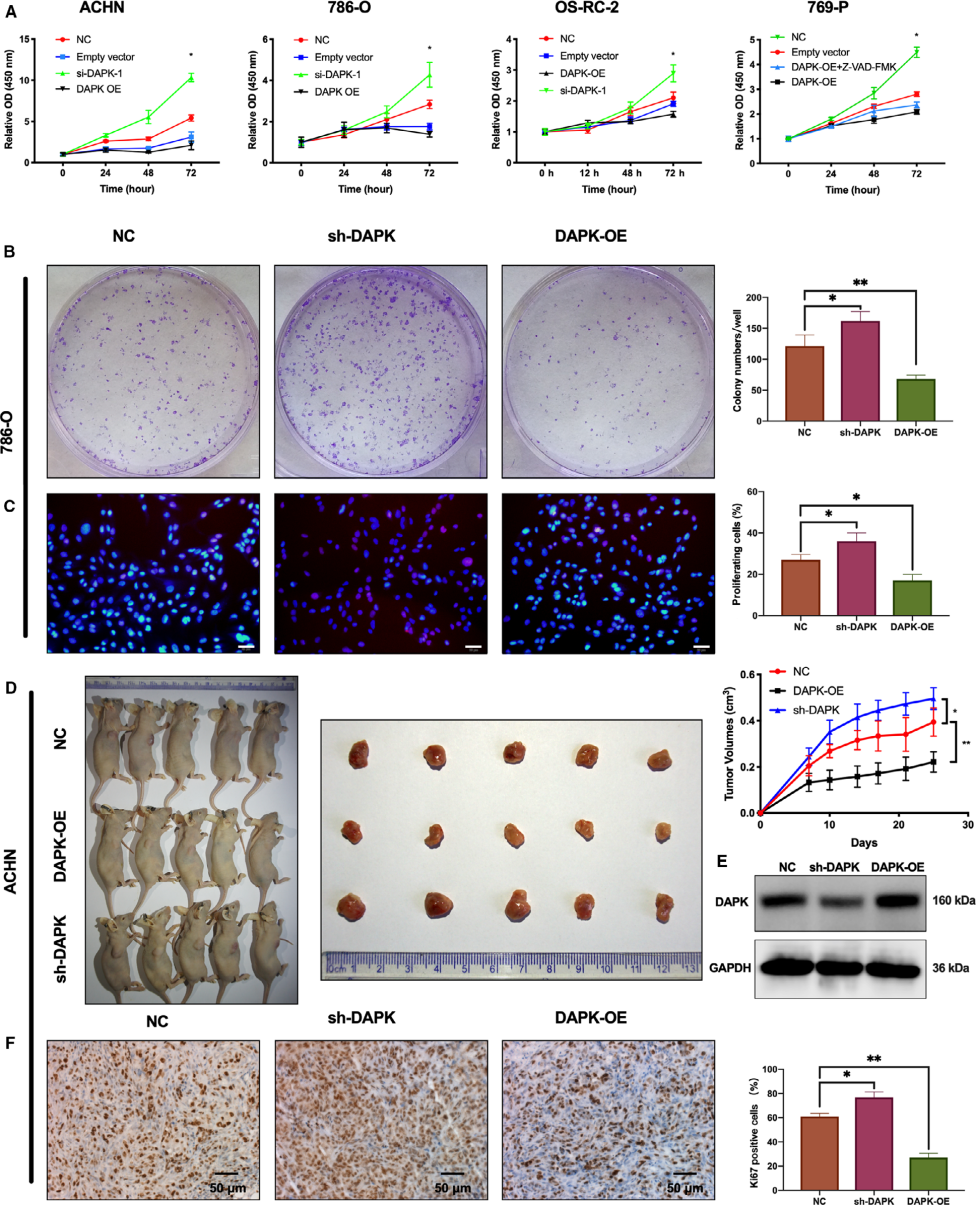
Impact of DAPK on the proliferation of renal cancer cells *in vitro* and *in vivo*. (A) CCK‐8 Kit was used to assess the growth of ACHN, 786‐O, OS‐RC‐2, and 769‐P after DAPK expression vectors or si‐DAPK transfection. Z‐VAD‐FMK (20 μm) was added to 769‐P cells 8 h before transfection to reduce the impact of apoptosis on cell growth. *P(one‐way ANOVA) < 0.05. Data are presented as mean ± SD (*n* = 3). (B) Colony formation of DAPK stable knockdown 786‐O cells, DAPK‐OE 786‐O cells, and control cells 10 days after seeding. *P(one‐way ANOVA) < 0.05, **P < 0.01. Data are presented as mean ± SD (*n* = 3). (C) Cell proliferation determined by EdU incorporation assay. Scale bar, 20 μm, *P (one‐way ANOVA) < 0.05, **P < 0.01. Data are presented as mean ± SD (*n* = 3). (D) The sacrificed mice and excised tumors were photographed. The tumor growth is represented by a line graph. *P (one‐way ANOVA) < 0.05, **P < 0.01. Data are presented as mean ± SD (*n* = 5). (E) Immunoblotting analysis of DAPK expression in excised tumors. (F) The IHC detection of Ki67 in control and treated tumors. The percentages of Ki67‐positive cells are presented as bar charts. Scale bar, 50 μm, *P (one‐way ANOVA) < 0.05, **P < 0.01. Data are presented as mean ± SD (*n* = 3).

The effect of DAPK on the proliferation of renal cancer cells was determined using CCK‐8, colony formation, and EdU staining assays. The caspase inhibitor Z‐VAD‐FMK was used when appropriate to reduce the proapoptotic effect of DAPK on renal cancer cells. CCK‐8 assays showed an inhibitory effect of DAPK on the growth of ACHN, 786‐O, OS‐RC‐2, and 769‐P cells (Fig. 3A). In the colony formation assay, cell colonies were stained and counted 10 days after plating 786‐O cells infected with sh‐DAPK vectors, DAPK‐overexpressing cells, and control cells. Cells with the stable interference of DAPK showed more cell colonies, whereas cells treated with DAPK overexpression had less cell colonies (Fig. 3B). Likewise, the EdU staining assay indicated that DAPK knockdown increased the percentage of EdU‐positive cells, whereas DAPK overexpression reduced the percentage of EdU‐positive cells (Fig. 3C).

*In vivo* xenograft tumor assays were also performed to validate the effect of DAPK on cell proliferation. As shown in Fig. 3D, tumors derived from the sh‐DAPK group grew faster than did tumors from the control group, while DAPK overexpression inhibited tumor growth. The mean tumor volumes of the control group, sh‐DAPK group, and DAPK‐overexpressing group were 394.1, 495.6, and 221.2 mm^3^, respectively. The expression of DAPK protein in transplanted tumors was validated by immunoblot (Fig. 3E). The IHC results showed the percentage of Ki67‐positive cells was highest in the sh‐DAPK group, while the percentage of Ki67‐positive cells was lowest in the DAPK‐overexpressing group (Fig. 3F).

### 3.4 DAPK inhibited the migration of renal cancer cells

**Figure 4:**
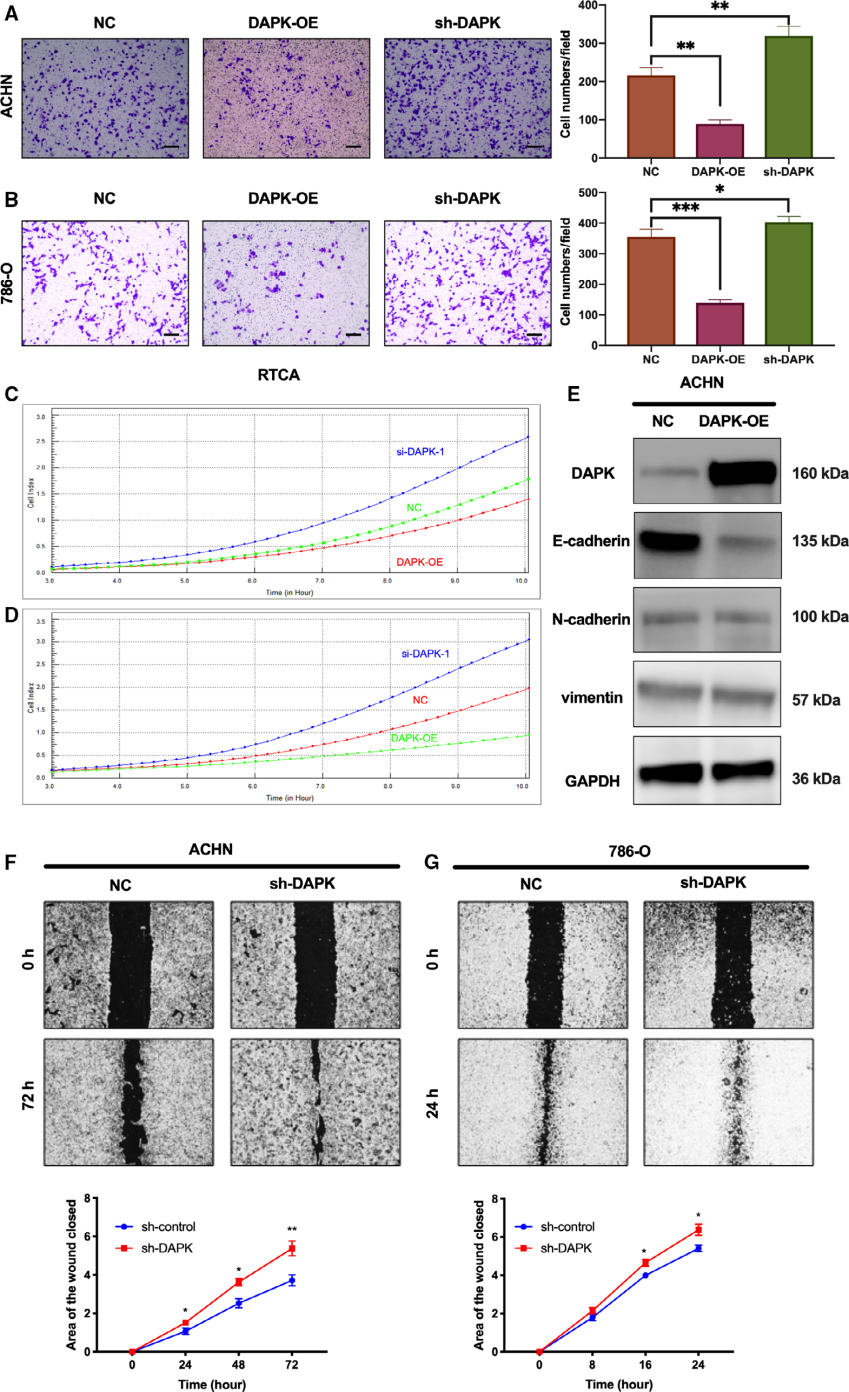
Effects of DAPK on renal cancer cell migration and expression of migration‐related proteins following DAPK overexpression. (A, B) Effects of DAPK on the migration of ACHN and 786‐O cells were analyzed by transwell assays. Cells were seeded in the upper chamber 24 h after transfection. Cells migrated to the lower surface of the membrane were stained and photographed under a microscope. Scale bar, 100 μm. The numbers of migrated cells per field were counted and shown as bar charts. *P (one‐way ANOVA) < 0.05, **P < 0.01, ***P < 0.0001. Data are presented as mean ± SD (*n* = 3). (C, D) Real‐time monitor of ACHN and 786‐O cell migration using xCELLigence RTCA DP system. (E) Impact of ectopic DAPK overexpression on the expression of E‐cadherin, N‐cadherin, and vimentin in ACHN cells. (F, G) Cell migration of ACHN and 786‐O cells was detected using a wound‐healing assay. *P (Student’s *t*‐test) < 0.05, **P < 0.01. Data are presented as mean ± SD (*n* = 3).

The effect of DAPK on the migration of renal cancer cells was also examined. Z‐VAD‐FMK was used to reduce the impact of apoptosis on cell migration. Transwell assays showed that more ACHN and 786‐O cells with DAPK interference were visualized on the lower surface of the transwell membrane 12 h after the cells were plated in the upper chamber (Fig. 4A,B). However, ectopic expression of DAPK inhibited the migration of renal cancer cells. Likewise, the results from RTCA, which monitored the migration of cells dynamically, indicated that ectopic expression of DAPK inhibited the migration of both ACHN and 786‐O cells to the lower surface of the chamber, and DAPK siRNA treatment promoted the migration of renal cancer cells (Fig. 4C,D). Since DAPK overexpression caused a massive detachment of cells, which caused problems for measuring the distance that the cells migrated, only cells with stable DAPK knockdown treatment were used in the wound‐healing assay. Of note, findings from the wound‐healing assay showed that stable DAPK knockdown promoted the migration of both 786‐O and ACHN cells irrespective of whether Z‐VAD‐FMK was used (Fig. 4F,G). Findings from immunoblotting showed DAPK overexpression caused a marked reduction in E‐cadherin expression in several renal cancer cell lines (Fig. 4E).

### 3.5 The ability of p53 to upregulate DAPK protein was suppressed in renal cancer cells

**Figure 5:**
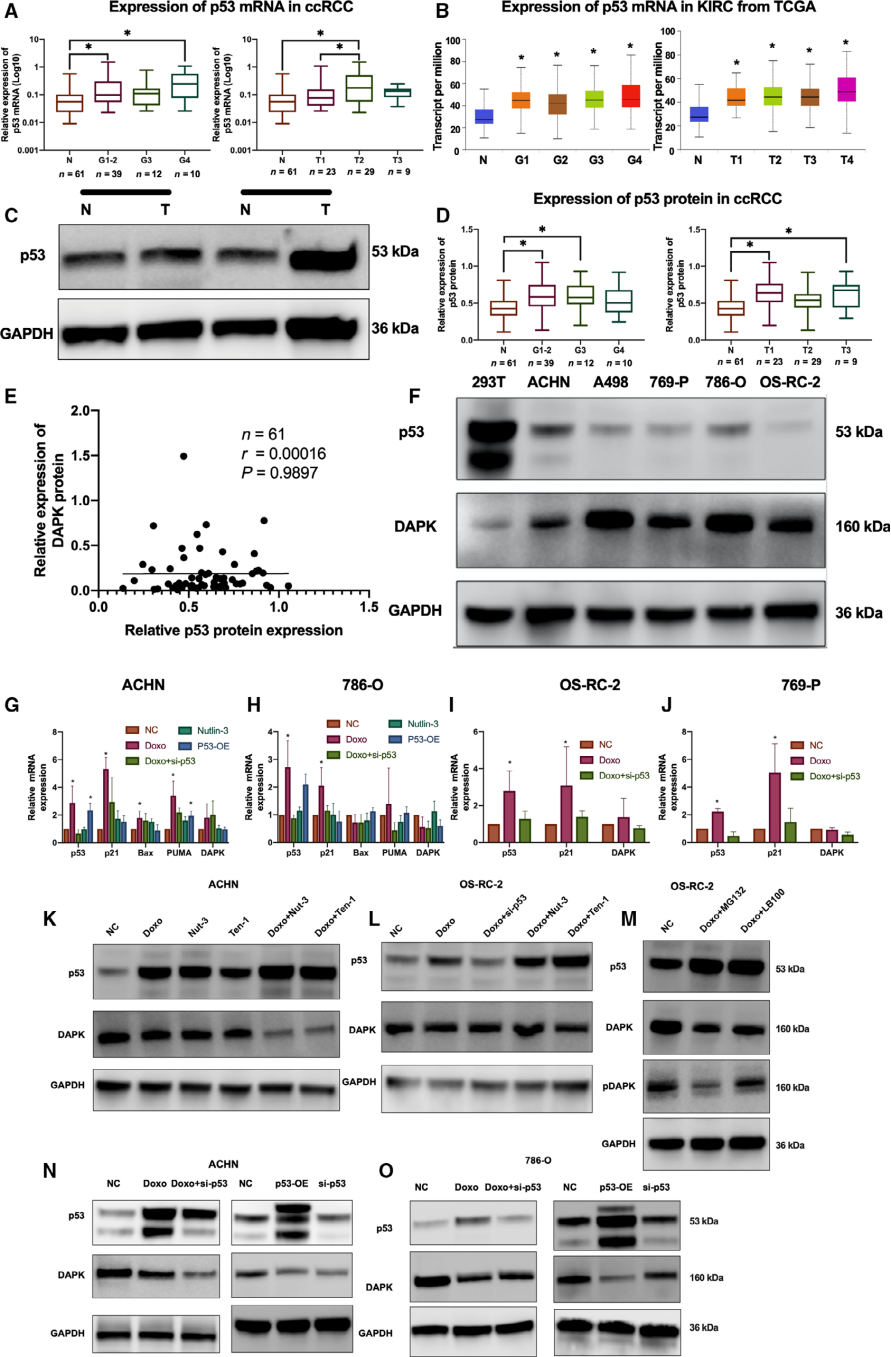
Expression of p53 in ccRCC tissue samples and renal cancer cell lines and correlation between p53 and DAPK expression. (A) Expression of p53 mRNA in normal renal tissues and ccRCC tissues shown as boxplots. *P (one‐way ANOVA) < 0.05. (B) Expression of p53 mRNA based on TCGA KIRC databases. (C) Immunoblot analysis of p53 expression in tumor tissues (T) and paired normal tissues (N) from ccRCC patients. (D) Relative p53 protein expression levels in human normal and renal cancer tissues shown as boxplots. *P (one‐way ANOVA) < 0.05. (E) Correlation between p53 protein expression and DAPK protein expression in human ccRCC tissue samples. Correlations were calculated according to the Pearson correlation. (F) Immunoblotting of p53 and DAPK in 293T cells and renal cancer cell lines. (G‐J) mRNA expression levels of p53 and p53 target genes following different treatments are presented as grouped column charts. *P (one‐way ANOVA) < 0.05. Data are presented as mean ± SD (*n* = 3). (K–O) Immunoblotting of p53 and DAPK expression following different treatments.

We first examined p53 expression on both the mRNA and protein levels in ccRCC and paired normal renal tissues using qPCR and immunoblotting. At the mRNA level, both G1‐2 and G4 ccRCC showed higher p53 mRNA levels than normal renal tissues (Fig. 5A). In addition, a significant difference was found between non‐neoplastic renal tissues and T1 stage tumors and between T1 and T2 stage tumors. Then, we examined p53 mRNA expression using the TCGA KIRC dataset, and a significant difference was observed between normal renal tissues and ccRCC samples of different grades or stages (Fig. 5B). Immunoblotting analysis showed p53 protein was higher in G1‐2 and G3 stage tissues than in normal renal tissues (Fig. 5C,D), and a significant difference was found between normal renal tissues and T1 tumors and between normal tissues and T3 tumors. We then examined the correlation between p53 and DAPK protein in ccRCC; however, no significant correlation was observed (Fig. 5E). The expression of p53 and DAPK in different renal cancer lines was also examined. The p53 and DAPK proteins can be detected in 293T and HK‐2 cells and all the renal cancer cell lines used in this study. However, basal levels of DAPK were not dependent on p53 protein levels (Fig. 5F).

Previously, DAPK was reported to be a transcription target of p53 protein in Saos‐2 and CNE1 cells (Luo *et al.*, 2011; Martoriati *et al.*, 2005). To determine whether p53 can induce DAPK transcription, different renal cancer cell lines were treated with DNA‐damaging chemotherapeutic drugs, p53 expression vectors, or p53 pathway activators. Doxorubicin (0.5 μg·mL^−1^) was quite effective in activating p53 among different DNA‐damaging agents in all renal cancer cell lines used (Fig. 5G–J). Although doxorubicin activated endogenous p53 and induced marked upregulation of several p53 target genes, including p21 and PUMA, in wild‐type p53 renal cancer cell lines, no comparable DAPK mRNA upregulation was observed. DAPK mRNA increased only slightly in ACHN and OS‐RC‐2 cells. To ensure that the upregulation of p53 target genes was specific to p53 activation, p53‐mutant 786‐O cells and p53 siRNA were used. Notably, p53 activation failed to induce the upregulation of the p53‐responsive genes Bax and PUMA in 786‐O cells (Fig. 5H). Moreover, pretreatment of renal cancer cells with p53 siRNA inhibited the upregulation of p53 target genes induced by doxorubicin. However, the kinetics of DAPK mRNA were not subjected to the same regulation by p53 activation or knockdown.

P53 expression vectors and p53 activators were also used to examine the effect of p53 on DAPK transcription. Although overexpression of wild‐type p53 expression vectors (pCMV6‐p53) induced the upregulation of p53 target genes such as p21 and PUMA, the increased p53 protein failed to induce the upregulation of DAPK mRNA (Fig. 5G–H). Likewise, although p53 activators nutlin‐3 (5 μm), tenovin‐1 (10 μm), or nutlin‐3/tenovin‐1 in combination with doxorubicin treatment increased p53 protein levels in renal cancer cell lines, they did not promote the upregulation of DAPK protein expression (Fig. 5K,L,N,O). To rule out the possibility that post‐translational regulations induced by p53 activation hindered DAPK protein upregulation, the protein degradation inhibitor MG132 (100 nm) was used to reduce DAPK degradation. Moreover, because the degradation of DAPK protein is usually preceded by DAPK dephosphorylation, the phosphatase inhibitor LB‐100 (4 μm) was also used in addition to doxorubicin. Despite the addition of MG132 or LB‐100, our findings indicated that p53 activation still failed to promote the upregulation of DAPK protein (Fig. 5M).

### 3.6 The p53‐regulated miR‐34a‐5p was found to target DAPK for translation inhibition

Previously, Martoriati and coauthors reported a p53 binding site in the first intron of the DAPK gene. This binding was also validated by our luciferase assay (data not shown). However, p53 overexpression or p53 activation increased the DAPK mRNA expression only slightly and failed to increase protein levels significantly. Thus, other regulatory mechanisms may be involved in inhibiting the upregulation of DAPK protein. We hypothesize that miRNAs may play a role in this suppression. Thus, we searched MirTarBase (http://mirtarbase.mbc.nctu.edu.tw/php/index.php) to look for miRNAs that could potentially suppress the upregulation of DAPK. Among these miRNAs, miR‐34a‐5p is specifically regulated by the functional p53.

**Figure 6:**
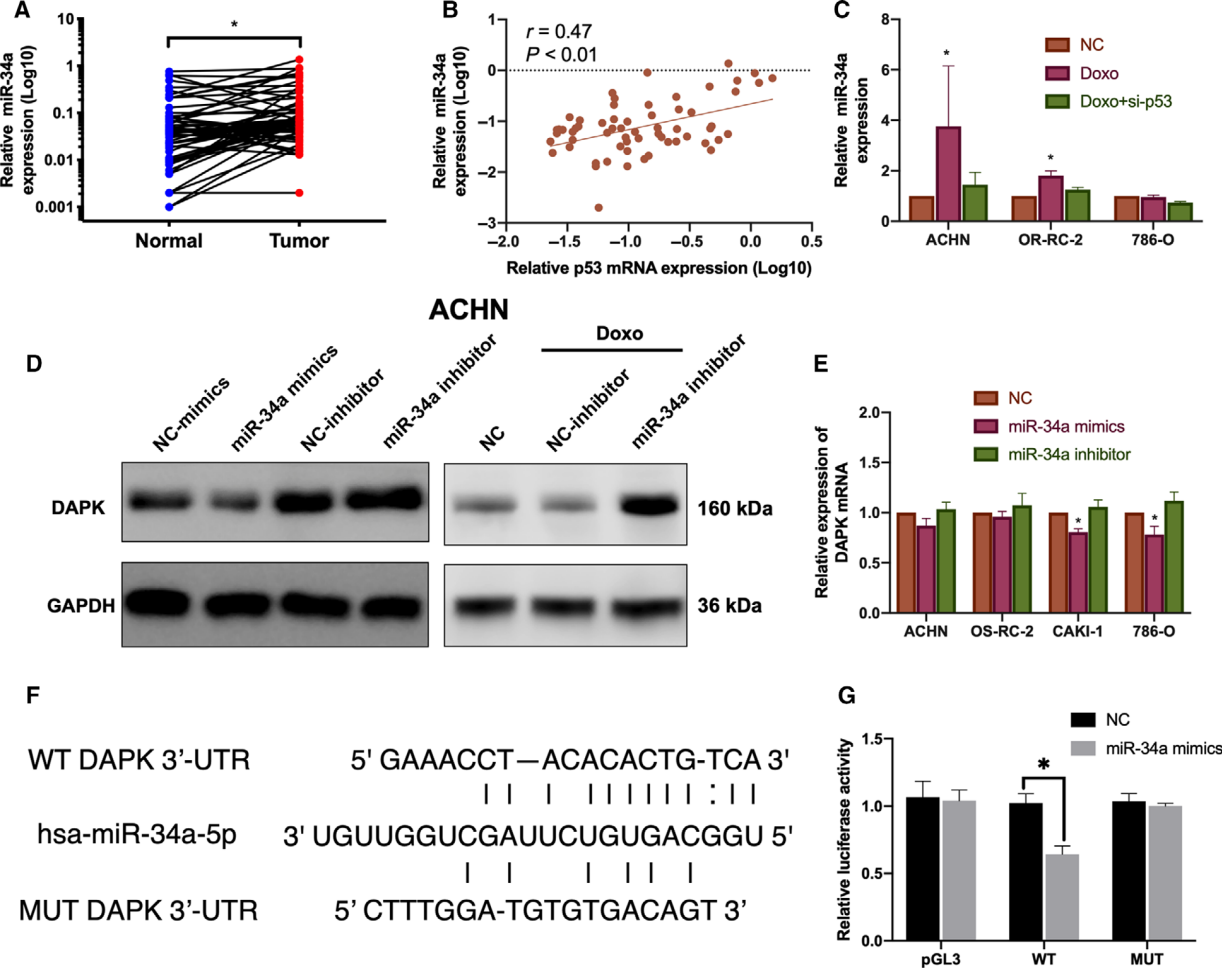
The p53‐regulated miR‐34a targets DAPK for translation inhibition. (A) Expression miR‐34a in ccRCC samples and paired normal renal tissues. *P (paired *t*‐test) < 0.05 (*n* = 61). (B) Correlation between p53 and miR‐34a expression in ccRCC tissues. Correlations were calculated according to the Pearson correlation. (C) Change in miR‐34a expression induced by doxorubicin, si‐p53 treatments, and both. (D) DAPK protein expression was regulated by miR‐34a mimics and inhibitor. (E) Changes in DAPK mRNA levels induced by miR‐34a mimics/inhibitor are presented as a bar graph. *P (one‐way ANOVA) < 0.05. Data are presented as mean ± SD (*n* = 3). (F) The predicted miR‐34a binding sequence in the 3'‐UTR of DAPK and mutant 3'‐UTR of DAPK. (G) The luciferase activities in 293T cells upon transfection of miR‐34a mimics and different reporter constructs are presented. *P (Student’s *t*‐test) < 0.05. Data are presented as mean ± SD (*n* = 3).

Initially, qPCR was used to measure the expression of miR‐34a‐5p in ccRCC tissues (Fig. 6A). Results from qPCR indicated that the expression of miR‐34a was higher in ccRCC than in normal renal tissues, although no significant difference was observed between ccRCC of different stages and grades. In addition, the expression of miR‐34a was positively correlated with p53 levels in ccRCC (Fig. 6B).

The ability of p53 to transactivate miR‐34a was then determined by qPCR (Fig. 6C). Treatment of ACHN and OS‐RC‐2 cells with doxorubicin increased miR‐34a expression, and the doxorubicin‐induced miR‐34a upregulation was inhibited by si‐p53 transfection. However, in p53‐mutant 786‐O cells, the expression of miR‐34a was not subjected to the same regulation.

We then examined the effect of miR‐34a on the expression of DAPK in renal cancer cell lines. Initially, ACHN was transfected with miR‐34a mimics and inhibitors. miR‐34a mimics lowered the DAPK protein levels compared to those in the control group, while miR‐34a inhibitors increased the DAPK protein expression (Fig. 6D). However, miR‐34a mimics and inhibitors failed to alter DAPK mRNA expression significantly in ACHN and OS‐RC‐2 cells (Fig. 6E). Slight downregulation of DAPK mRNA was only observed in CAKI‐1 and 786‐O following miR‐34a mimic treatment. Then, we treated renal cancer cells with doxorubicin or both doxorubicin and miR‐34 inhibitors, and we found that miR‐34a inhibitors rescued the ability of doxorubicin to upregulate DAPK protein. To investigate whether DAPK mRNA is a direct target of miR‐34a, reporter constructs containing the potential binding region of DAPK 3'‐UTR were generated (Fig. 6F). Cotransfection of miR‐34a mimics and DAPK 3'‐UTR constructs reduced the activities of DAPK reporters, whereas mutagenesis of the predicted miR‐34a binding sites failed to alter the reporter activities significantly (Fig. 6G). The findings from these studies indicated that miR‐34a can suppress the translation of DAPK protein by binding directly to the 3'‐UTR of DAPK mRNA.

### 3.7 Inhibition of miR‐34a enhanced the ability of p53 to induce cell death and suppress cell growth through upregulating DAPK

**Figure 7:**
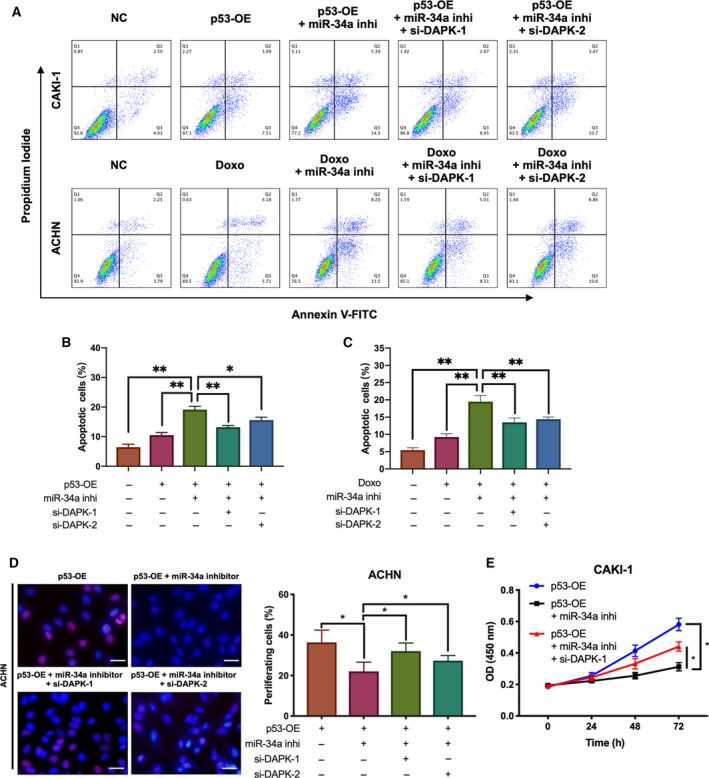
miR‐34a inhibition enhanced the tumor‐suppressive role of activated p53 via DAPK. (A–C) miR‐34a inhibitors increased apoptosis in renal cancer cells induced by p53 overexpression or p53 activation. *P (one‐way ANOVA) < 0.05, **P < 0.01, ***P < 0.001. Data are presented as mean ± SD (*n* = 3). (D) miR‐34a inhibitors enhanced the ability of p53 to suppress cell growth. Scale bar, 20 μm. (E) The CCK‐8 assay was performed to determine the effect of p53 and miR‐34a inhibitor on CAKI‐1 proliferation. *P (Student’s *t*‐test) < 0.05. Data are presented as mean ± SD (*n* = 3). (Doxo, doxorubicin; miR‐34a inhi, miR‐34a inhibitor).

To determine the role of miR‐34a in renal cancer cells, miR‐34 mimics and inhibitors were transfected into different renal cancer cell lines. Although the treatment altered the expression of DAPK protein, its effect on cell death and proliferation was nonsignificant. We then determined the role of miR‐34a in the p53‐DAPK axis in ccRCC. Apoptotic assays showed that miR‐34a inhibitors increased the percentage of cell death induced by doxorubicin or p53 overexpression (Fig. 7A–C). However, the addition of si‐DAPK rescued partially the increase in cell death. Similar results were also observed in CCK‐8 and EdU assays (Fig. 7D,E). miR‐34a inhibitors enhanced the ability of p53 to suppress cell proliferation; nevertheless, this suppression was weakened when si‐DAPK was used. These findings indicated that miR‐34a depended on DAPK to execute tumor‐suppressive function when p53 was activated.

### 3.8 AntagomiR‐34a acted in synergy with p53 to affect proliferation and apoptosis of tumors *in vivo*

**Figure 8:**
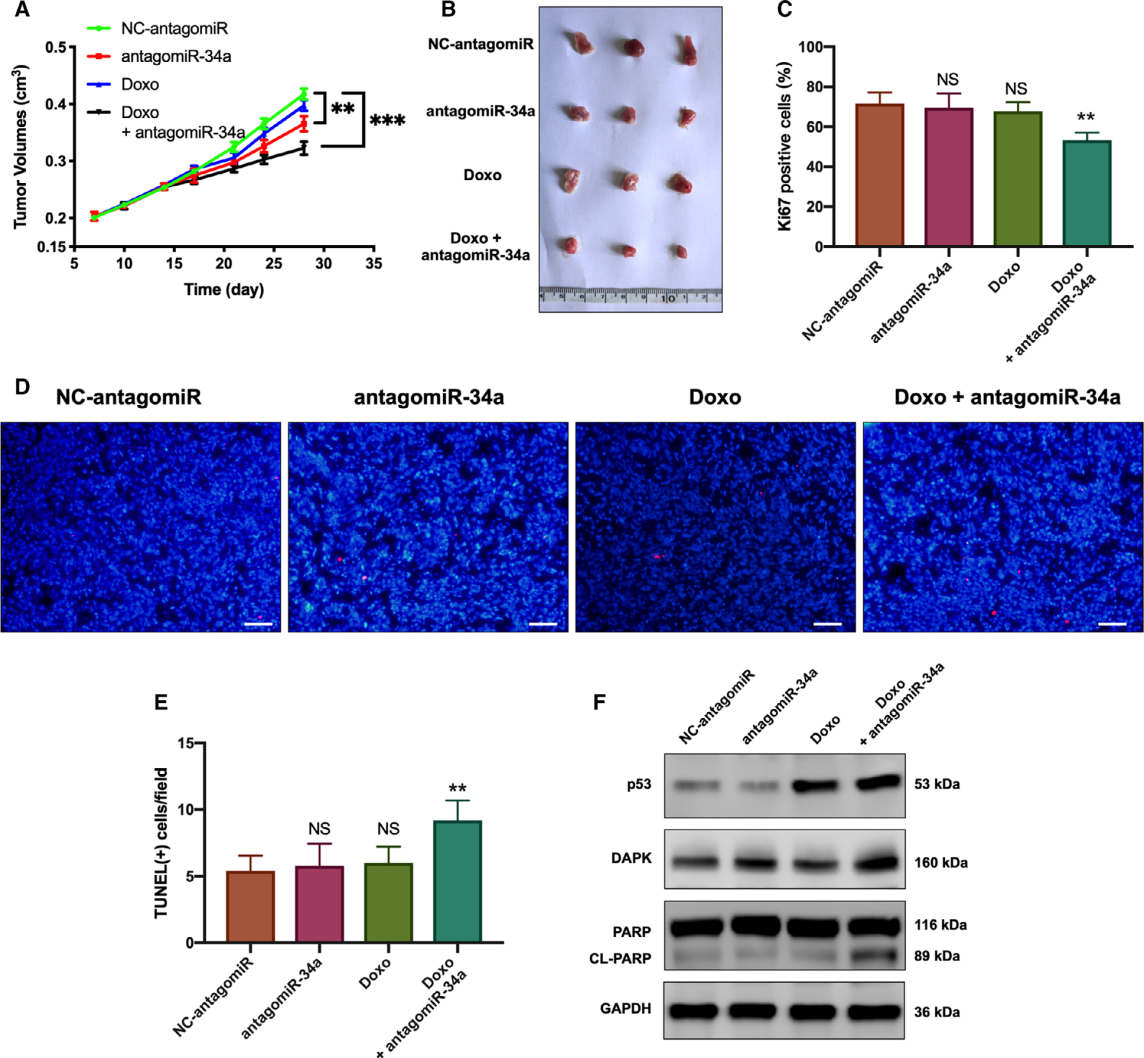
Effects of antagomiR‐34a inhibitor on ccRCC cell proliferation and apoptosis *in vivo*. (A) Tumor volumes of xenografts were measured at indicated time points and represented by a line graph. **P (Student’s *t*‐test) < 0.01, ***P < 0.001. Data are presented as mean ± SD (*n* = 3). (B) The excised tumors were photographed. (C) Percentages of Ki67‐positive cells in xenografts were analyzed by IHC staining and presented as bar charts. **P (Student’s *t*‐test) < 0.05. Data are presented as mean ± SD (*n* = 3). (C–E) Apoptosis of xenograft ACHN cells following different treatments was analyzed by TUNEL assay and presented as bar charts. *P (Student’s *t*‐test) < 0.05. Data are presented as mean ± SD (*n* = 3). Scale bar, 50 μm. (F) The expression of p53, DAPK, PARP, and cleaved PARP (CL‐PARP) in xenograft tumors was analyzed by immunoblot analysis. (Doxo, doxorubicin; miR‐34a inhi, miR‐34a inhibitor).

To determine the effect of miR‐34a‐5p *in vivo*, antagomiR‐34a, doxorubicin, or both were used to treat tumor xenografts in nude mice. Tumor growth curves were drawn twice weekly from day 7 to 28 (Fig. 8A). Tumors treated with NC‐antagomiR, antagomiR‐34a, doxorubicin, and antagomiR‐34a in combination with doxorubicin averaged 417.3, 372.0, 397.3, and 323.3 mm^3^, respectively (Fig. 8B). No significant difference was observed between the control group and the doxorubicin group. Although tumors treated with antagomiR‐34a were slightly smaller than tumors in the control group, the combined treatment inhibited the growth of tumor remarkably. The IHC results showed the percentage of Ki67 positively stained cells was lowest in the combined treatment group, while no significant difference was detected among other groups (Fig. 8C). Similar results were also found in TUNEL assays, where the combined treatment resulted in the highest percentage of TUNEL‐positive cells (Fig. 8D,E). Furthermore, immunoblot analysis of the excised tumors showed antagomiR‐34a with or without doxorubicin increased DAPK protein expression in xenograft tumors, and the combined treatment induced marked cleavage of the PARP (Fig. 8F).

## 4 Discussion

This study focused on ccRCC, the most common histologic subtype of RCC. Our data showed that DAPK mainly functioned as a tumor suppressor in ccRCC and that the expression of DAPK was reduced in certain subgroups of ccRCC. DAPK induced apoptosis and inhibited the growth of renal cancer cells both *in vitro* and *in vivo*. DAPK also affected the migration of renal cancer cells. Although DAPK was reported to be a direct transcriptional target of p53, in our study, no significant correlation between p53 and DAPK protein levels was observed in human ccRCC specimens or in renal cancer cell lines. Moreover, p53 activation failed to increase DAPK protein significantly. We hypothesized that miRNAs may play a role in this dysregulated p53‐DAPK signaling pathway and identified miR‐34a‐5p as a novel suppressor of DAPK translation. Meanwhile, miR‐34a was also a transcriptional target of p53. The upregulation of miR‐34a induced by activated p53 inhibited the translation of DAPK protein, thus compromising the tumor‐suppressive role of the p53‐DAPK pathway.

Although hundreds of papers on DAPK have been published over the past few decades, studies regarding the DAPK expression in RCCs are limited. The study by Nils Wethkamp et al focused on DAPK mRNA levels in RCCs, and no significant difference was found between ccRCC specimens of different stages (Wethkamp *et al.*, 2006). In the study by F Christoph et al, DAPK mRNA levels were found to be higher in renal tumors than in nontumor tissue from the same kidney. The expression of DAPK mRNA did not correlate well with tumor stage but was significantly lower in G3 tumors (Christoph *et al.*, 2007). Our findings are in part consistent with those of Christoph et al in that DAPK expression is reduced in high‐grade ccRCC; however, we did not observe a significant DAPK expression increase in ccRCC compared to that in the nontumor tissue on either the mRNA or protein levels. Caution should be exercised to compare the DAPK expression in ccRCC to normal renal tissues because they are of different cell compositions. Normal renal tissues include tubular epithelium, glomeruli, and other structures, while ccRCC originates from renal tubular epithelial cells. Our IHC results showed that DAPK protein is mainly detected in the cytoplasm of tubular epithelial cells; however, it is almost absent in renal glomeruli. Hence, it is not appropriate to compare normal renal tissues with ccRCC tissue samples.

The ability of p53 to induce DAPK transcription was originally reported by Martoriati in Saos‐2 cells (Martoriati *et al.*, 2005). Since then, DAPK has long been recognized as a direct transcription target of p53. However, findings from this study showed p53 protein levels did not correlate well with basal DAPK expression in either human ccRCC specimens or common renal cancer cell lines. Furthermore, p53 activation failed to upregulate DAPK protein expression. These findings suggested the ability of p53 to upregulate DAPK is not universally valid and has to be determined in a cancer‐type‐dependent manner.

An intact p53 pathway is essential for cells to undergo apoptosis or cell cycle arrest following DNA‐damaging therapies such as chemotherapy and radiotherapy. Although p53 is largely expressed as the wild‐type in ccRCC, ccRCC is highly resistant to chemotherapy and radiotherapy, suggesting that factors downstream of p53 may be abnormal in this process. Here, we found that p53‐DAPK dissociation contributed to the resistance of ccRCC to DNA‐damaging events and identified miR‐34a as the contributing factor for p53‐DAPK axis dysfunction.

Although most published studies have characterized miR‐34a as a tumor suppressor, some studies have reported a contrasting function of miR‐34a (Dutta *et al.*, 2007; Krause *et al.*, 2016; Pu *et al.*, 2017). In this study, DAPK was found to be a tumor suppressor in ccRCC; thus, miR‐34a played a tumor‐promoting role by inhibiting DAPK translation. In addition, the tumor‐promoting role of miR‐34a was only evident when the p53 was activated or upregulated, whereas in unstressed cells, its effect on apoptosis or cell growth was nonsignificant. These findings indicated that the role of miR‐34a played may vary depending on the target miR‐34a regulation and the cellular context.

## 5 Conclusions

Taken together, these results show that DAPK mainly functions as a negative regulator that acts downstream of p53 in ccRCC. The p53‐DAPK axis is suppressed due to increased miR‐34a‐5p, which is also induced by p53. miR‐34 inhibitors can rescue the disrupted p53‐DAPK pathway and be used as a potential therapeutic target to improve the treatment and prognosis of ccRCC patients.

## Conflict of interest

The authors declare no conflict of interest.

## Author contributions

Z‐FJ, C‐ZK, ZZ, and ZL conceived and designed the project; Z‐FJ and X‐TZ acquired the data; J‐BB, Z‐LL, and X‐KL included the patients; Z‐FJ, YZ, and JL interpreted the data; Z‐FJ and C‐ZK wrote the paper; C‐ZK, ZZ, and ZL supervised the study.

## Supporting information


**Fig S1.** Expression of DAPK in ccRCC.
**Fig S2.** Effects of DAPK on 769‐P apoptosis.
