# Supplementary material for: Inhibition of miR‐34a‐5p can rescue disruption of the p53‐DAPK axis to suppress progression of clear cell renal cell carcinoma
Source: Mol Oncol. 2019 Aug 24;13(10):2079–97. doi: 10.1002/1878-0261.12545 (PMC6763763; doi:10.1002/1878-0261.12545)
Supplement: Supplementary file 1 — Fig S1. Expression of DAPK in ccRCC. Fig S2. Effects of DAPK on 769‐P apoptosis. [file MOL2-13-2079-s001.docx]

Supplementary data


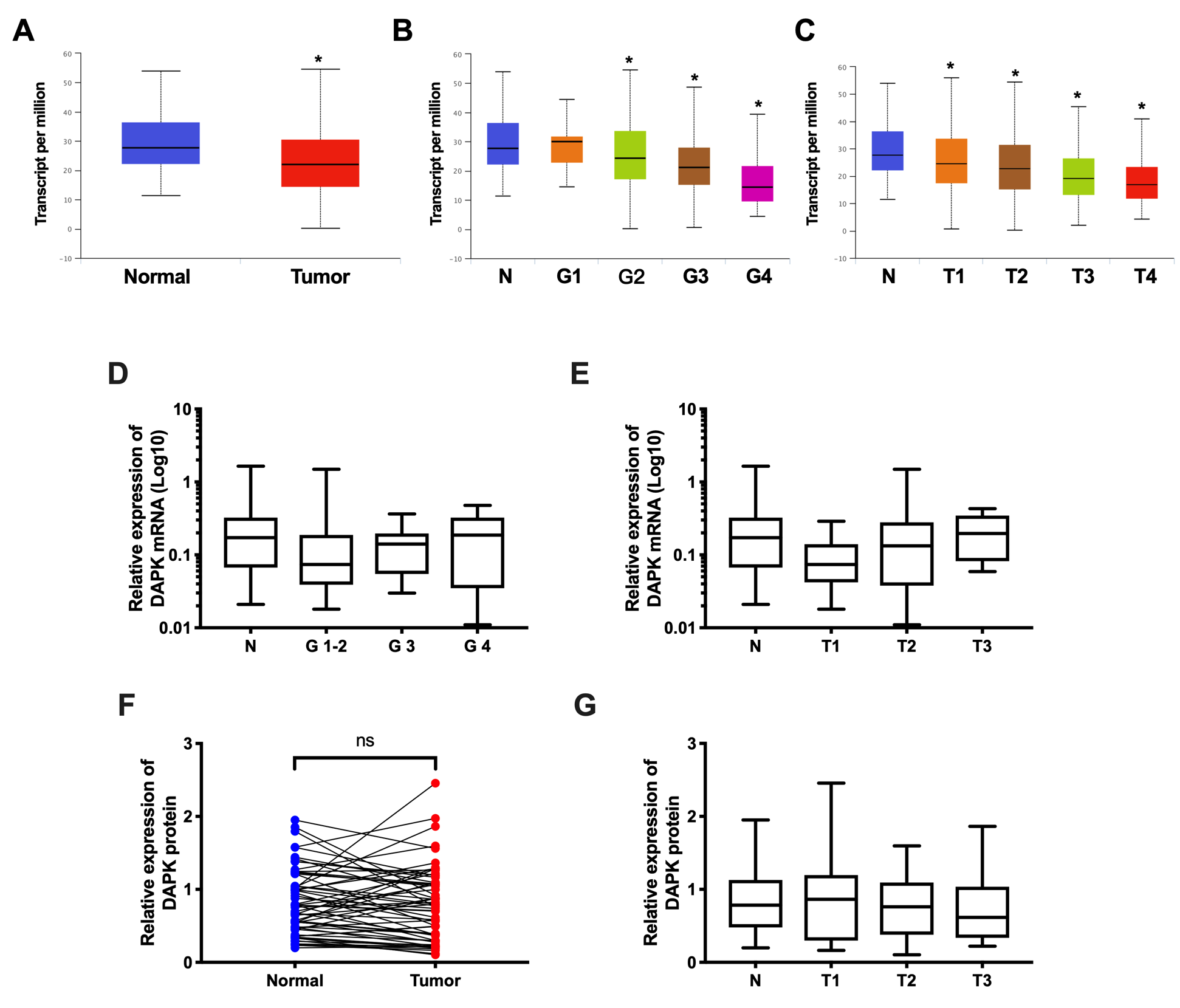


Figure 1 – Expression of DAPK in ccRCC.

(A-C) Expression of DAPK mRNA in normal renal tissues and ccRCC tissues of different grades and stages based on the TCGA KIRC dataset.

(D-E) qRT-PCR analysis of DAPK mRNA in normal renal tissues and ccRCC tissues of different grades and stages.

(F-G) Expression of DAPK protein in normal renal tissues and ccRCC tissues of different stages.


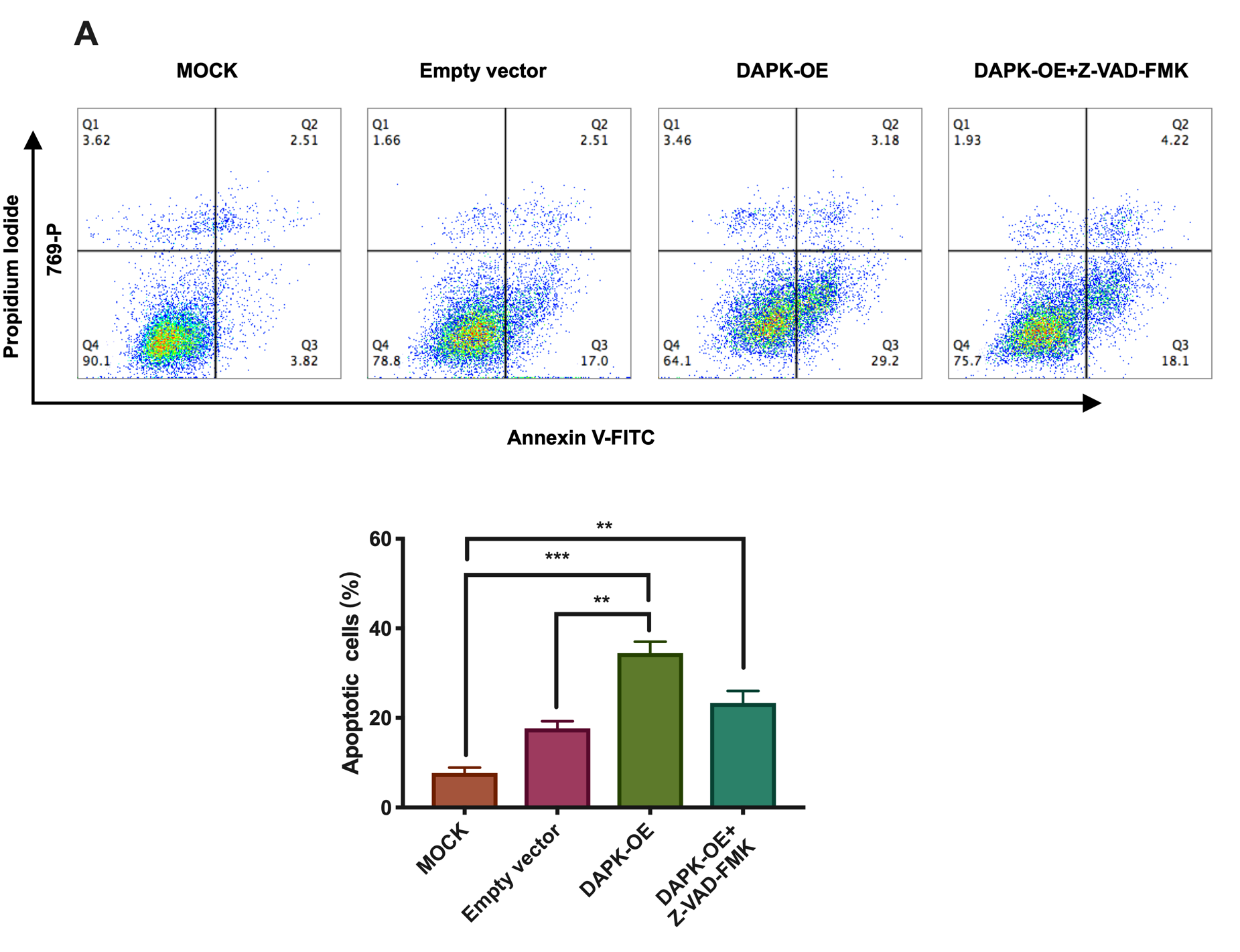


Figure 2 – Effects of DAPK on 769-P apoptosis.

(A) Effects of DAPK on 769-P apoptosis were analyzed by flow cytometry following Annexin V- FITC/Propidium iodide staining. In the MOCK group, only Lipofectamine 3000 and P3000 were added, while in the empty vectors group, pCMV3-untagged vectors were transfected. Pan-caspase inhibitor Z-VAD-FMK (20 μM) was used.
